# Multiple Levels of Influence on Older Adults’ Attendance and Adherence to Community Exercise Classes

**DOI:** 10.1093/geront/gnt075

**Published:** 2013-07-30

**Authors:** Helen Hawley-Hague, Maria Horne, Malcolm Campbell, Sean Demack, Dawn A. Skelton, Chris Todd

**Affiliations:** ^1^School of Nursing, Midwifery and Social Work, University of Manchester, UK.; ^2^Faculty of Development and Society, Sheffield Hallam University, Sheffield, UK.; ^3^School of Health, Glasgow Caledonian University, UK.

**Keywords:** *Instructor*, *Behavior*, *Attendance*, *Exercise programs*

## Abstract

***Purpose:*** To examine the influence of individual participant, instructor, and group factors on participants’ attendance and adherence to community exercise classes for older adults. ***Design and Methods:*** Longitudinal data from 16 instructors, 26 classes, and 193 older participants within those classes (aged 60–100 years) were examined. Data were collected using questionnaires on individual participants’ demographics, attitudes, health perceptions and conditions, and group cohesion. Instructors’ demographics, training, background, experience, attitudes, and personality were collected. Group factors included class type, cost, transport, and whether the class was held in an area of deprivation. Outcomes (attendance/adherence) were collected through attendance records. ***Results:*** Multilevel modelling (MLwiN) revealed both instructor and individual participant variables were important in understanding attendance and adherence. Individuals’ housing, education, mental well-being, group cohesion, and attitudes were important predictors of attendance at 3 and 6 months. Instructors’ age, gender, experience, and motivational training were important at 3 months, whereas instructor personality was important at both 3 and 6 months. Having attended longer than 6 months at baseline, participants’ attitudes, weeks offered, instructors’ personality, and experience were associated with adherence at 6 months. ***Implications:*** Results suggest that instructors’ characteristics alongside individual participant factors play a role in influencing participants’ attendance to exercise classes. These factors should be considered when setting up new programs.

Promoting physical activity among older adults is an important public health issue (Nelson et al., 2007). In later life, exercise brings physiological and psychological benefits, reducing illness, improving functional ability, and well-being (American College of Sports Medicine, 2009).

Despite all known health benefits of exercise, only 30% of those aged 65+ report any regular exercise (Agency for Healthcare Research and Quality, 2002). Even when older adults initiate exercise, they often discontinue involvement within 6 months of starting a program (Jancey et al., 2007). The literature suggests that it is in the first 6 months that an older adult commits to attending a class (Stigglebout, Hopman-Rock, Crone, Lecher, & Van Mechelen, 2006). Therefore, it is likely that attitudes of older adults change during this period, depending on whether expectations are met. This suggests the first 6 months is a particularly important stage in attendance. Once there is commitment to attend the class, ill-health appears to be the dominant reason for drop out (Phillips et al., 2010).

Older adults’ uptake and adherence to exercise classes revolves around factors such as attitudes, expectations, and whether expectations are fulfilled (Hays, Pressler, Damsuh, Rawl, & Clark 2010; Yardley et al., 2006). Self-efficacy (belief in ability to carry out the task) is a significant predictor of exercise in older adults; if they have been active in the past, they are more likely to have stronger self-efficacy (Rhodes et al., 1999). The Theory of Planned Behavior (TPB) has been particularly useful for assessing older adults’ attitudes in relation to exercise uptake and adherence (Lucidi, Grano, Barbaranelli, & Violani, 2006; Yardley, Donovan-Hall, Francis, & Todd, 2007). It has also been used to examine instructors’ attitudes about older adults’ participation in exercise classes (Hawley, Skelton, Campbell, & Todd, 2012). TPB is based on three main concepts: (a) perceived behavioral control (PBC), (b) attitudes (outcome expectations), and (c) social influences (Ajzen, 1988). It has been argued that the constructs of PBC and self-efficacy are interchangeable (Ajzen & Driver, 1992). Therefore, following Ajzen and Driver (1992), we consider PBC to be “the perceived ease or difficulty of performing the behavior and it is assumed to reflect past experience as well as anticipated impediments and obstacles” (p. 208). The second concept, attitude, concerns the advantages and disadvantages of a particular behavior (outcome expectations) and, when considered in relation to exercise maintenance, can include how closely those outcome expectations are met. The third concept, social influence, includes several constructs, subjective norms (perceived beliefs of other people, e.g., family), perceived social support (support from others for behavior), and modelling (following observed behavior of others). The core of TPB is the individual’s intention to perform behavior, the stronger the intention, the more likely the action will occur (Ajzen & Driver, 1992). The three elements of TPB are important in influencing intention, as confirmed by a number of exercise studies among older adults (Dean, Farrell, Kelley, Taylor, & Rhodes, 2007; Rhodes et al., 1999).

Social support links to older adults’ attitudes (social influence), increases self-efficacy, and promotes long-term adherence (McAuley, Jerome, Elavsky, Marquez, & Ramsey, 2003). Social support can be provided through verbal encouragement to attend a class by family member, peer, or exercise instructor (McAuley et al., 2003), the support of the group (encouragement to attend as a social occasion), or taking participants to classes (Hedley, Suckley, Robinson, & Dawson, 2010; Stathi, Mckenna, & Fox, 2010). Motivational interviewing has also been found to influence older adults’ attitudes toward exercise (Greaney et al., 2008), which is something that could be provided by health professionals, families, peers, or instructors. Leadership and group support have been reported by older adults as important for adherence (Stathi et al., 2010). However, there is limited quantitative literature and we do not yet understand the different ways in which group cohesion and instructors influence participant adherence.

Using a longitudinal cohort study, instructors, their existing classes, and participants were followed for a 12-month period to investigate how instructor, group, and individual characteristics influence older adults’ attendance and adherence to exercise classes. We report up to 6-month data here. We predicted that:

1. Older adults’ attendance and adherence to exercise classes would be associated with individual participant, group, and instructor characteristics.
2. Positive attitudes held by older adults’ and instructors’ about participation in the exercise class would be associated with both higher attendance and adherence.
3. Participants who had been attending the class for longer than 6 months at baseline would be more likely to adhere.

## Methods

### Participants and Recruitment

An existing cohort of instructors (Hawley et al., 2012) from across the United Kingdom was followed up. All eligible instructors (*N* = 62) were approached and asked to participate via letter. The inclusion criteria included class delivery in a community venue (different locations across central/northern England) and that they were considering establishing new classes (so as to recruit participants new to the class). All classes were multicomponent exercise classes (e.g., aerobic, strength, balance, and stretching) for older adults, were offered once a week, and comprised a range in the number of classes offered over the 6-month period (Table 1). For clarity, we refer to the older people participating in exercise classes in our study as “participants” and the instructors as “instructors.” The inclusion criterion for participants of the exercise classes was that they must be aged ≥60 years. We did not exclude participants with cognitive impairments, but all participants were able to complete questionnaires unaided. Instructors first approached their class participants for initial consent, and the researcher then attended classes to recruit participants (*N* = 361). Baseline data collection was carried out between May and August 2010. In total, 16 instructors, 27 classes, and 200 participants agreed to participate. Only one class approached failed to complete the questionnaires. Seven participants were excluded as they were under 60 years, leaving 26 classes and 193 participants.

**Table 1.** Descriptive Statistics—Baseline Variables for Class Participants

Class participants (*N* = 193)	Counts (%) (unless stated otherwise)
Gender	Women: 175 (90.7%)
Age	Mean: 76.1 (*SD* 7.8); range: 60–100
Ethnicity	White British: 182 (94.3%)
Education	Age left school: mean 15.2 (*SD* 1.2)
	No education since school: 114 (62.0%)
Economic factors	Owner of own home: 141 (73.1%)
Medical conditions related to	Nervous system: 57 (29.5%)
	Circulatory: 81 (42.0%)
	Musculoskeletal: 92 (47.7%)
	Respiratory: 37 (19.2%)
	Endocrine/metabolic: 40 (20.7%)
Participants attending class less than 6 months at baseline	47 (24.4%)
Attitudes (AFRIS score)	Mean: 36.35 (*SD* 3.6); range: 19–42
Group cohesion (PAGEQ scores)	ATG-T—mean: 7.66 (*SD* 1.1); range: 3.67–9.00
	ATG-S—mean: 7.68 (*SD* 1.2); range: 2.83–9.00
	GI-T—mean: 7.20 (*SD* 1.2); range: 4.20–9.00
	GI-S—mean: 7.64 (*SD* 1.4); range: 2.25–9.00
SF12 physical (PCS)	Mean: 38.24 (*SD* 11.6); range: 9.94–62.33
SF12 mental (MCS)	Mean: 50.72 (*SD* 9.8); range: 17.02–70.98
Does home exercise	165 (85.5%)
Instructor encourages home exercise	162 (83.9%)
Number of classes offered over 6 months follow-up	Mean: 21.58 (*SD* 2.3); range: 16–24

Questionnaires were used with class participants at baseline, 3 months, and 6 months; questionnaires were administered to collect instructor data at baseline only. Group characteristics were provided by instructors, along with attendance records for the full 6-month period. Ethical approval was granted by University of Manchester Committee on the Ethics of Research on Human Beings.

### Measures

#### Instructor Questionnaire.

##### Demographics.

Inst‑ ructors’ demographic information (Table 3) was taken from our previous study (Hawley et al., 2012) and updated for training. All instructors had to have obtained a “Level 3” older adult’s exercise qualification or higher from an accredited provider; the nature of these qualifications and providers are described elsewhere (Hawley et al., 2012). Instructors were also asked whether they had undertaken motivational training, as previously we found this related to more positive attitudes about older adults’ participation in classes (Hawley et al., 2012).

**Table 3.** Descriptive Statistics—Baseline Variables for Instructor Characteristics

Instructors (*N* = 16)	Counts (%) (unless otherwise stated)
Gender	Women: 14 (87.5%)
Age	Mean: 54.5 (*SD* 12.6); range: 29–75
Ethnicity	White British: 15 (93.8%)
Training qualifications	EXTEND: 10 (62.5%)
	EXTEND and PSI: 3 (18.8%)
	PSI: 1 (6.2%)
	EXTEND and YMCA: 1 (6.2%)
	Otago: 1 (6.2%)
	Motivation training: 8 (50.0%)
Background	NHS clinical: 1 (6.2%)
	Sports and fitness: 5 (31.3%)
	Education: 2 (12.5%)
	Community: 4 (25%)
	Social care: 1 (6.2%)
	Other: 3 (18.8%)
Experience (length of experience using delivery in months)	Mean: 53 (*SD* 34.5); range: 3–120
Attitudes (amended AFRIS score)	Mean: 71.88 (*SD* 6.5); range: 63–89
Personality scores (Saucier’s mini markers)	Extraversion—mean: 6.34 (*SD* 1.3); range 4.38–8.25
	Agreeableness—mean: 8.13 (*SD* 0.7); range 6.63–9.00
	Conscientiousness—mean: 7.28 (*SD* 0.7); range 5.50–8.63
	Emotion stability—mean: 6.70 (*SD* 1.1); range 4.38–8.88
	Intellect—mean: 5.89 (*SD* 1.1); range: 4.75–7.75

*Notes:* The different qualifications enable the instructor to deliver different types of exercise delivery, for example, EXTEND is exercise to music and PSI is falls prevention exercise. Please see Hawley and coworkers (2012) for further explanation.

##### Instructors’ attitudes.

Based on previous work (Hawley et al., 2012), we investigated instructors’ attitudes to older adults’ participation in exercise classes using an amended version of the 6-item TPB-based Attitudes to Falls-Related Interventions Scale (AFRIS) (Yardley & Todd, 2008). We added an additional PBC question (“Older adults are capable of participating in a ‘mostly seated/mostly standing’ class”) because of limitations to the wording of the original PBC question (Hawley et al., 2012). We used an amended version of the identity question from the validated AFRIS (“I think that an older adult would feel that they are the kind of person who should attend a ‘mostly seated/mostly standing’ class”) even though it had been removed during analysis in our previous study (Hawley et al., 2012) as it increased Cronbach α. We present a combined instructor attitudes score for easier comparisons with participant AFRIS (Yardley & Todd, 2008). Overall the instructor AFRIS includes 15 questions (Cronbach α = 0.68). The present sample is too small for robust results, but in our previous study with a larger sample, Cronbach was α = 0.80 (Hawley et al., 2012).

##### Instructors’ personality.

Data have not previously been collected on personality, but research with older adults suggests it could be important (Loughead & Carron, 2004). Saucier’s “mini markers” are based on the big five personality traits: (a) extraversion, (b) agreeableness, (c) conscientiousness, (d) emotional stability, and (e) intellect. They form a relatively short scale and have excellent validity and reliability (Saucier, 1994). Each personality trait has a corresponding scale. Extraversion was found to have Cronbach α = 0.84, agreeableness Cronbach α = 0.85, conscientiousness Cronbach α = 0.82, emotional stability Cronbach α = 0.79, and intellect Cronbach α = 0.74, indicating good reliability of scales.

#### Participant Questionnaire.

##### Demography, health, and well-being.

Data on gender, ethnicity, date of birth, and how long the participants had been attending the classes (weeks) were collected, so we could establish how many participants had already been attending for longer than 6 months when we started data collection. Information about health conditions was provided by participants and based on ICD-10 codes, for example, diseases of the nervous system (WHO, 2013). Older adults self-reported their mental and physical health status (MCS and PCS, respectively) using the SF12 questionnaire (Ware, Kosinski, & Keller, 1996); PCS had Cronbach α = 0.89 and MCS had Cronbach α = 0.80.

##### Participants’ attitudes.

Older adults’ attitudes about attending the class were explored using the original 6-item AFRIS (Yardley & Todd, 2008). This asks about attitudes, social influences, PBC, intention, and identity. Although identity is not part of TPB, it was previously shown to be an important predictor (Yardley & Todd, 2008). AFRIS had Cronbach α = 0.70.

##### Group cohesion.

Class and group cohesion were investigated using the 21-item PAGEQ (Estabrooks & Carron, 1999, 2000). PAGEQ has four subscales scored on 9-point scale: (a) *individual attractions to the group-task* (ATG-T; individual’s perceptions of their involvement in the group task), (b) *individual attractions to the group-social* (ATG-S; individual’s perceptions of acceptance and interaction with the group), (c) *group integration-task* (GI-T; individual’s perceptions of the group and how it bonds around the collective task), and (d) *group integration-social* (GI-S; individual’s perceptions of how the group bonds as a social entity). The PAGEQ subscales had Cronbach α values as follows: ATG-T, 0.94; ATG-S, 0.94; GI-T, 0.85; GI-S, 0.77.

#### Group Measures.

Characteristics of classes were provided by instructors and in the analysis, we refer to them as group measures (Table 2), including whether transport was provided, participants charged, the venue was open to the public or referral only, and class type (mostly seated or mostly standing, see Hawley et al., 2012). As we did not have access to individuals’ addresses, we assessed deprivation using an ecological measure, the Index of Multiple Deprivations, which gives a deprivation score based on a variety of deprivation indicators (Office of National Statistics, 2011).

**Table 2.** Descriptive Statistics—Baseline Variables for Group Characteristics

Group factors (*N* = 26)	Counts (%) (unless otherwise stated)
Number of mostly seated classes included in the study	21 (80.8%)
Number of classes which charge participants	21 (80.8%)
No transport provided	23 (88.5%)
Open class (not referral)	19 (73.1%)
Deprivation score (IMD score)	Mean: 24.45 (*SD* 14.2); range: 2.5–54.79

#### Outcome Measures.

In previous studies, the terms “adherence” and “attendance in weeks” are used interchangeably (Hughes et al., 2006; Sjosten et al., 2007), causing lack of clarity in how the two concepts are defined. Thus, we differentiate between attendance to the class and adherence to the overall regimen. Attendance is a simple count of the number of classes attended, whereas adherence represents the longer term commitment to the class and is an indicator of drop-out.

##### Attendance in weeks.

Weekly class attendance records provided by the instructor were collected at 3- and 6-month follow-up points. Attendance details in weeks were calculated from the attendance records and indicates whether the participant attended each week the class was offered. We refer to this as “attendance” in the analyses presented.

##### Adherence.

Adherence levels were calculated at each follow-up period. Nonadherence was defined as “those not attending at follow-up and have not attended for 4 weeks, and have not given a reason for nonattendance or those who have stated they are dropping out.” In our analyses, we refer to this as “adherence.”

### Statistical Analysis

Data were analyzed using SPSS V15.0 (SPSS Inc., 2006). MLwiN was used for multilevel modelling (Rasbash, Charlton, Browne, Healy, & Cameron, 2009). Investigation of the relationships between instructor, group, and individual participant measures implies a data hierarchy resulting in a “nested” data structure (Peugh, 2010). This three level structure violates the independence assumption required by traditional multiple regressions (Peugh, 2010) and thus multi-level modelling is appropriate (Kreft & De Leeuw, 1998).

The number of class weeks offered was used in the multiple regressions on the individual participant level to control for differences in attendance in weeks caused by variation in what was offered. Percentage of weeks as the outcome variable was considered, but because of limited variability in weeks offered in the first 3 months, it was not possible to analyze the variable as ordinal data. Thus, multilevel linear multiple regression was the most appropriate method for both 3- and 6-month attendance, providing the most information about the model (Rasbash et al., 2009). Multilevel multiple logistic regression was most appropriate for the adherence model (Rasbash et al., 2009).

Before analysis, a null model for attendance was tested in MLwiN to assess the appropriateness of modelling the hierarchical structure (Rasbash et al., 2009). A null model contains only a response variable and no explanatory variables apart from a constant and is used at baseline for estimation of explained versus unexplained variance (Kreft & De Leeuw, 1998). Although the natural structure of the data suggested a three level structure, based on the null model, we decided to analyze the data using a two level model (instructor and individual participant) as there was not enough variance at group level. There was not sufficient variance in instructor background, ethnicity, or training nor participant ethnicity to permit meaningful analysis so these variables were excluded. For multilevel multiple linear regressions, we used a maximum likelihood method, the iterative generalized least squares regression (Goldstein, 1995).

When testing the null model in MLwiN for adherence, we found that no variance was explained on the instructor level. This appears to be due to lack of variance in the outcome measure. Therefore, single level multiple logistic regression in SPSS was used with individual participant and instructor variables.

We checked for assumptions made in all three models; the residuals for the flat models were approximately normal and homogeneity of variance was found to be acceptable. There was no evidence of multicollinearity (tolerances all ≥0.10) in any models; thus, it is reasonable to assume that if the assumptions for the flat model are not strongly violated, then they are unlikely to be violated in the multilevel models.

Predictors were initially considered for regression modelling if they showed an individual association with an outcome at a conservative level of significance (*p* ≤ .25) (Hosmer & Lemeshow, 2000). As recommended by Rasbash et al. (2009), all continuous variables included in the multilevel models were centered on the grand mean. In the final models, we only entered variables with *p* ≤ .10 and a final *p* ≤ .05.

#### Missing Data.

Only participants with full data set of all included variables were included in multiple regression models. One instructor did not complete the personality questions so her participants were not included in the final model. One class had a change of instructor in the early stages of data collection. The new instructor’s characteristics were used in the final models. To consider the impact of missing data on these models, we compared key variables at baseline for those included in the analysis and those excluded. Those participants excluded from the final model were not significantly different from those included, in attendance (13.7 vs. 15.1, *t* = 1.76, *df* = 186, *p* = .08), nor drop-out (58.8% vs. 41.2%, χ^2^ = 0.97, *df* = 1, *p* = .33). This suggests that results using a reduced sample are not likely to differ from the full sample.

## Results

### Participants

We recruited 16 (25.8%) instructors, 26 (96.3%) classes, and 193 class participants; based on class attendance records, we recruited 53.5% of 361 eligible class participants. All 193 class participants completed baseline questionnaires; 126 class participants completed 3-month questionnaires, and 109 participants completed 6-month questionnaires. One instructor chose to opt her participants out of completing questionnaires after baseline but continued providing attendance records. Attendance records were available for 189 participants from baseline to 6 months (four participants unidentifiable). Most instructors were women (*N* = 14, 87.5%), with mean age 54.5 (*SD* 12.6; range 29–75). There was a wide range in experience of instructors, but the majority (87.5%) were EXTEND trained and had positive attitudes toward participation of older adults in exercise classes (Table 3). Most class participants were women (*N* = 175, 90.7%), mean age = 76.1 (*SD* 7.8), age range 60–100, and had long-term conditions (Table 1). Forty-seven (24.4%) participants had been attending the class for less than 6 months at baseline. The mean number of classes attended by participants during the 6 months was 14.76 (*SD* 5.1). At 6 months, 17 (8.8%) participants had dropped out of classes and 4 were lost to follow-up.

### Attendance in First 3 Months

#### Null Model.

We tested the null model for baseline to 3-month attendance. For the three level model, 73% of the variation in participant attendance was found at Level 1 (individual participant), only 0.03% at Level 2 (group), but 23% at Level 3 (instructor). Therefore, the group did not seem to explain variation in attendance. There was little ability to detect variability at this level because of small numbers. Thus, variation in outcome is located primarily at individual participant level, although the instructor does play a role; therefore, a two level model was adopted. The final two level null model of baseline to 3-month attendance accounted for 73% variance at individual participant level and 27% variation at instructor level. The final model (Table 4) accounted for 23% of the 73% of variance identified by the null model at individual participant level and 100% of the 27% of variation identified at instructor level. This leaves no variance left to be explained at instructor level, but 50% of the potential variance at individual participant level is left unexplained by the final model.

**Table 4.** Attendance in Weeks: MLwiN (2 Level) Model, Nonsignificant Variables Removed at *p* < .10

	Baseline questionnaire to 3-month attendance (*N* = 132 participants and *N* = 14 instructors)			Baseline questionnaire to full 6-month attendance (*N* = 131 participants and *N* = 14 instructors)		
	*B* (*SE*)	CI		*B* (*SE*)	CI	
Individual variables						
Housing	−1.180 (0.536)*	−2.230	−0.129	−2.940 (0.928)*	−4.759	−1.121
Education						
Age left school	0.168 (0.187)*	−0.198	0.534	0.263 (0.354)*	−0.431	0.957
Education since school	0.796 (0.449)**	−0.084	1.676	0.912 (0.844)**	−0.742	2.566
SF12 physical	—			0.057 (0.035)	−0.012	0.126
SF12 mental	−0.012 (0.023)**	−0.057	0.033	−0.005 (0.043)*	−0.089	0.079
Cohesion						
ATG-S	0.321 (0.237)*	−0.143	0.464	0.699 (0.442)**	−0.167	1.565
GI-T	−0.343 (0.234)**	−0.801	0.116	−0.494 (0.448)**	−1.372	0.384
AFRIS (Attitudes)	0.166 (0.073)**	0.022	0.309	0.400 (0.137)**	0.131	0.668
Do you carry out exercises at home?	0.867 (0.505)	−0.123	1.857	—		
Number of classes offered	0.240 (0.237)	−0.224	0.704	0.505 (0.183)*	0.146	0.864
Instructor variables						
Gender (female)	−4.396 (1.307)**	−6.958	−1.834			
Age	−0.139 (0.071)*	−0.278	0.000			
Experience (in months)	0.053 (0.019)*	0.016	0.090	0.021 (0.011)	−0.001	0.042
Motivational training	2.631 (1.003)*	0.665	4.597	1.601 (0.820)	−0.006	3.208
Personality						
Extraversion	−2.455 (0.875)*	−4.170	1.715	−1.381 (0.443)*	−2.492	−0.513
Agreeableness	−3.804 (1.175)*	−6.107	−1.501			
Conscientiousness	1.746 (0.585)*	0.599	2.892	2.811 (0.670)**	1.498	4.124
Emotional stability	0.749 (0.437)	−0.107	1.605			
Intellect	−0.554 (0.263)*	−1.069	−0.038			

*p < .05. **p < .001.

### Individual Participant Variables Included in Multivariate Model

In the first 3 months, owning one’s own home had a significantly negative association with attendance compared with renting (*p* < .05). Leaving school older (*p* < .05) and completing further education (*p* < .001) were positively associated with attendance. Class participants with higher SF12 mental health (MCS) composite scale scores were less likely to attend (*p* < .001). Class participants with higher PAGEQ ATG-S scores (more positive perceptions of their social interaction with the group) had higher class attendance (*p* < .05). Class participants with higher PAGEQ GI-T scores (more positive perceptions of how the group bonded around doing the exercises) had lower class attendance (*p* < .001). Class participants with higher AFRIS scores (more positive attitudes toward participating in an exercise class) had higher class attendance (*p* < .001; Table 4).

#### Instructor Variables.

Female instructor gender was negatively associated with participants’ class attendance (*p* < .001). Instructor age was also negatively related to participant attendance (*p* < .05), although instructor years of experience was positively related to participant attendance (*p* < .05). Instructors who had undertaken motivational training were more likely to have participants with higher class attendance (*p* < .05). Analysis of personality variables revealed instructors who had more “extravert,” “agreeable,” or “intellectual” traits had lower participant attendance (*p* < .05), whereas those with “conscientious” traits had higher class attendance (*p* < .05).

### Attendance for the Full 6 Months

#### Null Model.

To ensure consistency with the model reported earlier, we examined a two level model (individual participant and instructor) to investigate baseline to 6 months class attendance in weeks. The null model accounted for 79% of variance at individual participant level and 21% of variation at instructor level. The final model accounted for 21% of the 79% of variance identified at individual level and 100% of the 21% of variation identified at instructor level. This leaves no variance left to be explained at instructor level, but 58% of potential variance at individual participant level is not explained by the final model.

#### Individual Participant Variables.

As with the 3-month analysis, over 6 months, owning one’s own home had a significantly negative association with class attendance compared with renting (*p* < .05). Leaving school at an older age (*p* < .05) and completing further education (*p* < .001) had positive effects on attendance.

Class participants with higher SF12 MCS scores were less likely to attend the class frequently (*p* < .05). Class participants with higher PAGEQ ATG-S scores (more positive perceptions of their social interaction with the group) were more likely to attend (*p* < .001), whereas those with higher PAGEQ GI-T scores (more positive perceptions of how the group bonded around exercises) were less likely to attend (*p* < .001). Class participants with higher AFRIS scores had higher attendance (*p* < .001). Perhaps unsurprisingly, the more weeks offered the higher number of weeks attended (*p* < .05).

#### Instructor Variables.

Only instructor’s personality was significantly related to attendance over the full 6 months. Instructors who had more “extravert” personality traits had participants with poorer attendance (*p* < .05), whereas those with “conscientious” traits were more likely to have participants with higher attendance (*p* < .001).

### Adherence

#### Null Model.

To ensure consistency, we tested a two level null model using MLwiN for the adherence outcome but found no variance explained at instructor level (Table 5). Single level multiple logistic regression in SPSS was used with individual participant and instructor variables. Variance explained by the final model was pseudo-*R* ^2^ = .34 (Menard, 1995).

**Table 5.** Adherence at 6 Months Using SPSS Multiple Logistic Regression With Nonsignificant Variables Removed at *p* < .10

	*B* (*SE*)	Baseline questionnaire to 6-month adherence (*N* = 146)		
		OR	CI	
			Upper	Lower
Individual variables				
Less than 6-month class attendance at baseline	−1.593 (0.807)*	0.20	0.042	0.990
SF12 mental	0.084 (0.049)	1.09	0.988	1.196
Cohesion				
ATG-S	0.677 (0.375)	1.87	0.944	4.103
GI-T	—	—	—	
AFRIS (attitudes)	0.229 (0.109)*	1.26	1.015	1.558
Number of classes offered	0.371 (0.157)*	1.45	1.066	1.970
Instructor variables				
Experience (in months)	−0.043 (0.016)*	0.96	0.928	0.989
Personality				
Conscientiousness	1.583 (0.701)*	4.87	1.233	19.244

*p < .05.

#### Individual Participant Variables.

Participants who had attended the class for <6 months at baseline were less likely to adhere at 6 months (*p* = .05). Higher SF12 MCS (*p* = .09) and higher PAGEQ ATG-S scores (more positive perceptions of social interaction with the group) (*p* = .07) although related to adherence were not significant. Higher AFRIS scores were significantly related to adherence (*p* < .05), as was number of classes offered (*p* < .05).

#### Instructor Variables.

Instructor experience was negatively associated (*p* < .05), and instructor conscientiousness (*p* < .05) was positively associated with adherence.

## Discussion

Instructor as well as participant characteristics play a role in influencing participants’ attendance to exercise classes. Participants’ attitudes, perceptions about group cohesion, and instructor variables such as personality traits emerged in all three of the final models in relation to attendance and adherence. Some variables only related to either attendance or adherence (i.e., participant education level), indicating that these two variables measure different concepts, and although a participant may not attend every week, they could be ultimately committed to the class.

Instructors’ attitudes do not seem to influence participants’ class attendance or adherence. This could be due to a lack of differences in training between instructors as different training has shown to relate to attitudes (Hawley et al., 2012). There was lack of variance at instructor level in the adherence model, this seems unlikely to be because the instructor does not have an influence, but rather because the variation was too small (i.e., there were too few participants not adhering, and lack of differences between instructors). This is the same for group level variables across all three models and so the relationship with both attendance and adherence outcomes could not be explored. Therefore, although instructors do have some influence, the role of the instructor and group in promoting attendance and adherence is still not clearly established.

Surprisingly, home ownership was associated with poorer levels of attendance. This variable acts as a proxy for social economic status. Higher education levels and affluence are normally associated with higher levels of exercise as indicated by home ownership. However, in this study, it seems affluence is associated with having other commitments. This was mentioned by both instructors and participants with affluent participants taking long vacations.

Whether the participant had been attending the class for less than 6 months at baseline was significantly related to whether they dropped out (odds ratio = 0.20). This concurs with literature which suggests it takes 6 months for behavior to be adopted (Stigglebout et al., 2006) and therefore drop-out in the first 6 months is more likely to occur. It is also supported by feedback from instructors who said that if participants were going to drop-out, they did so in the initial stages, those who stayed adhered long term.

Positive mental well-being (SF12) at baseline negatively affected attendance but was positively related to adherence. Previous research has reported that poorer mental health negatively affects activity levels (Rogerson, Murphy, Bird, & Morris, 2012). On the other hand, exercise classes have been shown to provide social contact, improved mood, and reduced stress levels (Phillips et al., 2010; Sjosten et al., 2007).

The class participants’ ATG-S scores increased over the first 3 months and were positively associated with attendance and adherence. This supports previous research that focuses on the importance of group cohesion and the class as a social occasion (Estabrooks et al., 2004; Tulle & Dorrer, 2012). Class participants’ perceptions of how the group bonds around exercise (GI-T scores) were negatively related to attendance, although previous research reports a positive relationship (Estabrooks & Carron, 1999). Furthermore, neither scores on ATG-T (participant’s perception of their own involvement in group task) nor GI-S (perceptions of how the group bonded as a social entity) were related to attendance or adherence. Further research on the stability and utility of PAGEQ is recommended because one would have predicted positive associations with each subscale.

Class participants’ attitudes toward the class were positively related to attendance and adherence to the class, supporting previous evidence about the relationship between attitudes and adherence (Lucidi et al., 2006). It could be that instructors empowered participants, increasing confidence in ability to perform the exercises, therefore improving attendance/adherence levels. McAuley et al. (2003) found greater support in the exercise setting related to greater exercise effect, self-efficacy, and higher participation levels.

The number of classes available for participants during the 6-month period was related to attendance and adherence. Some classes were cancelled because of instructors’ holidays and exceptionally harsh winter weather during the study period. However, data collection was over the same winter period for all classes, so all classes were disrupted by poor weather. It is not surprising that nondelivery of classes relates to attendance as some participants had the opportunity to attend significantly more classes than others; however, there is little literature on this.

For instructors, being older or women was associated with lower attendance of participants. However, there were only two men in the sample with relatively small numbers of participants attending their classes, so this finding should be considered with caution. The relationship between instructor age and lower attendance cannot currently be explained and needs further exploration. The experience of the instructor had a positive relationship with attendance in the first 3 months of the study. However, it was not significantly related to attendance over 6 months and experience had a negative relationship with adherence at 6 months. Instructor experience could be important in ensuring participants feel comfortable in the first 3 months of attending a class and may be important in building self-efficacy, but not all participants were new to the class. It is not clear why instructor experience had a negative relationship with participant adherence; a previous study (Seguin et al., 2010) established instructors’ experience could have a role to play in adherence, which requires further exploration.

Instructors who had undertaken motivational training were likely to have participants who attend more frequently, significantly so in the first 3 months. Qualitative feedback from instructors suggests this is the stage where participants are encouraged to stay and when their motivational role could be key. Instructor personality is significantly related to attendance. Being too extravert, agreeable and coming across as too intelligent, is associated with poorer class attendance. On the other hand, conscientiousness is positively associated with attendance and adherence to the class and perhaps relates to person-centered delivery and meeting expectations. Previous qualitative research provides some limited support for the role of instructors’ personality, particularly having a sensitive and patient approach (Stathi et al., 2010). However, instructor personality has the potential not only to influence participants’ attendance directly but through influence on participants’ attitudes, delivery of the class, and cohesion of the group. Further exploration is required to establish the relationships between personality and attendance.

## Limitations

There are limitations to the study. The majority of participants and instructors recruited to the group were white Caucasian, limiting generalizability. The relatively small numbers of instructors recruited means that findings need to be considered with caution. Instructors and class participants who agreed to participate in the study are likely to be enthusiasts, introducing potential bias. Approximately half of the potential participants were recruited; this potential self-selection bias may explain why there was low drop-out from classes.

There were issues with missing data and representation of new participants (attended the class for less than 6 months at baseline). However, this study was pragmatic in intension and worked with existing classes. To recruit enough new participants to follow from first attendance would have meant recruiting only newly trained instructors with new classes, which itself would have introduced bias as experience of instructor is related to participant adherence (Seguin et al., 2010). To maximize generalizability, we recruited a variety of instructors and classes across a broad area of England. However, the majority of the instructors had EXTEND, PSI, or Otago qualifications, or a combination of these. Only one instructor had the YMCA qualification and none of the other recognized qualifications (e.g., Medau, KFA, and Laban) are represented. This may have affected the instructors’ attitudes and approach to their classes. As illustrated in previous research (Hawley et al., 2012), EXTEND instructors had more positive attitudes about older adults’ participation in mostly seated classes than instructors with other qualifications. However, instructors’ attitudes were not found to relate to attendance and adherence nor to participants’ attitudes in this study. Not all the qualifications held by instructors are available in other countries, but instructors’ training was not included in the final models due to lack of variation.

## Recommendations and Conclusion

Some factors that influence attendance and adherence cannot be changed, such as participant demographics. However, the instructor does play a role in influencing participants’ attitudes and a role in managing and meeting expectations. Participants’ attitudes about the class are important to both attendance and adherence. It is, therefore, important that classes meet participants’ expectations and instructors give positive physical (e.g., improved function) and mental (e.g., improved mood) outcomes. Goal setting could be a way to show participants how the class has met their expectations and instructors may find it beneficial to attend motivational training to assist with this and with dealing with barriers (Evers, Klusmann, Schwarzer, & Heuser, 2012). Social influence/outcomes were found to be important and are reflected in the relationship between participants’ attitudes, group cohesion, and attendance and adherence. It is recommended that the instructor fosters group cohesion and promotes peer support both in the class and through additional social events.

We found that the number of classes offered were important in influencing adherence. Therefore, it is recommended that instructors try to ensure that someone else is able to deliver their class if they are unable to do so. When new classes are started (before behavior is established), the use of an experienced instructor may promote attendance, or new and established instructors should deliver together until experience is gained.

Further studies looking at attendance and adherence should consider carefully the definition and scope of measures selected, as the literature provides no clear consensus on how adherence should be measured. Although this study did not focus on rehabilitation classes, its findings have implications for delivery in these settings. Studies of community- based falls rehabilitation reveal that patients followed up in the community after rehabilitation, deteriorate almost back to pre-rehabilitation state (Hawley, 2009). There are opportunities to explore the influence of individual, group, and instructor variables on rehabilitation classes. Larger exploratory studies are required to further explore the role of the instructor and the group in older adults’ adherence to general exercise and rehabilitation classes.

## Funding

This work was supported by a Medical Research Council (MRC) Doctoral Training Grant (MR/K500823/1) and the University of Manchester through the Faculty of Medical and Human Sciences.
